# The impact of stress on the transcriptomic signature of iNKT1 cells

**DOI:** 10.1016/j.bbrep.2021.101163

**Published:** 2021-10-29

**Authors:** Georgia Papadogianni, Inga Ravens, Ahmed Hassan, Oliver Dittrich-Breiholz, Günter Bernhardt, Hristo Georgiev

**Affiliations:** a Institute of Immunology, Hannover Medical School, 30625, Hannover, Germany; b Research Core Unit Genomics, Hannover Medical School, 30625, Hannover, Germany

**Keywords:** iNKT cells, Stress response, Cell isolation, Transcriptome, P2X7

## Abstract

Invariant natural killer T (iNKT) cells develop in thymus before emigrating and settling peripheral tissues and organs. In contrast to regular naïve T cells, most iNKT cells do not continuously recirculate but are rather sessile and can adopt phenotypically as well as functionally to their tissue environment. To explore this in more detail, we focused on the most widely distributed CD4^+^iNKT1 cells and compared the transcriptome of cells isolated from liver and spleen. Whereas there are only very few genuine differences in the transcriptomes of CD4^+^iNKT1 cells of these two organs, the mode of cell isolation left clear marks in the transcriptomic signature. In contrast to liver cell isolated in the cold, cells prepared by enzymatic tissue digestion upregulated quickly a series of genes known to respond to stress. Therefore, to avoid erroneous conclusions, a comparison of expression profiles must take into consideration the history of cell preparation.

## 1 Introduction

Invariant natural killer T (iNKT) cells represent a small subset of specialized T cells. They express a narrow spectrum of T cell receptors recognizing foreign or self-glycolipids presented by the MHCI-like receptor CD1d [1,2]. iNKT cells are considered innate-like cells because they express markers distinguishing antigen-experienced T cells and produce cytokines very quickly upon challenge. These features place them at the interface between innate and adaptive immunity and numerous reports document their multifaceted roles in immune-relevant processes (see Ref. [3] for an excellent review). iNKT cells originate in thymus and are selected into the iNKT developmental pathway at the DP stage starting a stepwise maturation program [4,5]. Early studies in mouse suggested that already immature iNKT cells can leave thymus and continue to mature into sessile iNKT cells in peripheral tissues and organs [[6], [7], [8]]. But recent results implied that also mature iNKT cells can emigrate from thymus [9,10]. Instrumental towards a better understanding of the iNKT cell biology was the recent development of a classification system that combined expression of key transcription factors important for iNKT cell differentiation and function with a set of surface markers as well as the preferential production of landmark cytokines IL4, IL17 and IFNγ. Three main subsets were defined [11,12]: (CD122/CXCR3)^+^CD4^+/−^PLZF^lo^TBET^+^RORγt^−^ iNKT1 cells with predo-minating IFNγ-secretion, (CD122/CXCR3)^−^CD4^+^PLZF^hi^TBET^−^RORγt^-^ iNKT2 cells producing mainly IL4 and (CD122/CXCR3)^−^CD4^−^ PLZF^int^TBET^-^RORγt^+^ iNKT17 cells secreting IL17.

Previous analyses indicated that iNKT cells of thymus, liver and spleen largely express the same repertoire of genes [13,14]. Considering that all iNKT cells are thymus-born, it is conceivable to assume that such differences reflect functional adaptation to the particular micro-milieu and needs of the hosting organ [15,16]. In this study, we compared the transcriptomes of CD4^+^iNKT1 cells isolated from liver and spleen. Except for very few genes, the transcriptomes were nearby identical when cells were isolated at low temperatures prior to RNA isolation. However, when liver cells were obtained following an enzymatic treatment of tissue, this had profound effects on both, the general yield of cells and the transcriptome signature. We provide evidence that temperature during cell isolation is a main factor causing the observed effects.

## 2 Materials and methods

### 2.1 Animals

C57BL/6 N (BL6) and BALB/cAnNCrl (BALB/c) mice were bred in the animal facility of Hannover Medical School or purchased from Charles River Laboratories. Keeping and usage of animals as cell donors was done according to MHH guidelines and approved by LAVES, Lower Saxony. All animals analyzed were 8–12 weeks old. Female mice were used in the case of transcriptomic analyses. For all other experiments male and female mice served as cell donors.

### 2.2 Cell suspensions

Thymi and spleens were cut into small pieces, meshed through 40 μm cell strainers and washed once in FACS buffer (PBS with 3% FCS). All steps were performed in the cold.

For the isolation of liver cells, mice were perfused with cold PBS or with cold PBS supplemented with 10 μM A-438079 (Sigma). Following harvest, livers were split in two halves and the weight of each half was measured. Next, one half of each liver sample was further cut into small pieces and digested with 0.5 mg/mL collagenase D (Roche) and 0.025 mg/ml DNase I (Roche) in RPMI 1640/10% FCS for 30 min at 37 °C (liver_H_). After digestion, EDTA was added at final concentration of 20 mM. Samples were meshed through 40 μm cell strainers and washed with RPMI 1640/10% FCS. Alternatively, the second half of each liver samples were cut into small pieces and meshed through 40 μm cell strainers without digestion in the cold (liver_C_). To obtain lymphocytes, samples in 40% Percoll were layered on 70% Percoll and centrifuged at 20 °C for 20 min at 2000 rpm.

### 2.3 Antibodies and flow cytometry

Following block with 3% rat serum, cells were incubated with anti-mouse antibodies in FACS-buffer. Antibodies used were: anti-CD122 PE (TM-β1), anti-CD122 FITC (TM-β1), anti-CXCR3 PE (CXCR3-173), anti-CXCR3 FITC (CXCR3-173), anti-CD4 PerCP (RM4-5), anti-CD4 BV510 (RM4-5), anti-CD8a BV421 (53–6.7) and anti-TCRβ PECy7 (H57-597) all from Biolegend, anti-CD138 BV421 (281–2) from BD Biosciences, anti-CD44 PE (IM7.8.1), anti-CD25 Alexa 488 (PC61 5.3), anti-CD45R (B220) eFluor 450 (RA3-6B2) and anti-CD45R APC-eFluor 780 (RA3-6B2) from Invitrogen, anti-CD62L Pacific Orange (made in house), anti-CD3 AF488 (17A2, made in house) and CD1d tetramer loaded with PBS57.

Data were acquired on LSR II (BD) and analyzed using FlowJo software (Treestar).

### 2.4 Annexin V apoptosis assay

Single-cell suspension from spleen samples were prepared in RPMI 1640/10% FCS or in RPMI 1640/10% FCS supplemented with 10 μM A-438079 (Sigma). The isolated cells were plated into 96-well plates at a density of 2x10^6^ cells/200μl/well and incubated for 1h at 37 °C. Following incubation, spleen and liver samples were stained for iNKT cell surface markers along with Annexin V (Annexin V Apoptosis Detection kit, BD bioscience) and were finally resuspended in Annexin V binding buffer/DAPI (0.1 μg/ml).

### 2.5 Cell sorting and RNA isolation

Cells from spleen and liver were pooled from 4 to 6 female BL6 or BALB/c mice. Liver cells were prepared either in the cold (liver_C_) or at 37 °C (liver_H_) as described above. CD4^+^iNKT1 cells were sorted at the FACSAria Fusion (BD) as B220^−^CD3^+^tet^+^CD122^+^CD4^+^ cells from thymus and as CD138^-^B220^−^CD3^+^tet^+^CXCR3^+^CD4^+^ cells from spleen and liver. CD138 was included as an additional marker to exclude plasma B cells from the sorted populations. RNA was isolated using the RNeasyPlus Micro Kit (Qiagen).

### 2.6 Real time PCR

Spleen and liver cells were prepared under cold conditions. cDNA was synthesized using Super Script II Reverse Transcriptase (Invitrogen). Real time PCR was performed with SYBR Green Premix Ex Taq II (Takara) and primers specific for murine *Hprt* or *Hspa1a*.

*Hprt* forward: 5′-AGTGTTGGATACAGGCCAGAC-3′

*Hprt* reverse: 5′-TTCAACTTGCGCTCATCTTAGG-3′

*Hspa1a* forward: 5′-ACTGACAGCGGAGACTCTGC -3.

*Hspa1a* reverse: 5′-ACTGAACACATGCTGGTGCTG -3′

### 2.7 Library generation, sequencing and raw data processing

*Library generation, quality control, and quantification:* 1–5 ng of total RNA were used for library preparation with the ‘SMARTer Stranded Total RNA-Seq Kit v2 – Pico Input Mammalian’ (Takara/Clontech). Generated libraries were barcoded by dual indexing approach and were finally amplified by 11 cycles of PCR. Quantification of libraries was performed by use of the ‘Qubit® dsDNA HS Assay Kit’ (ThermoFisher Scientific).

*Library denaturation and Sequencing run:* Equal molar amounts of 12–15 individually barcoded libraries were pooled for one sequencing run. The library pool was prepared according to the Denature and Dilute Libraries Guide (Illumina). 1.3 ml of denatured pool was loaded on an Illumina NextSeq 550 sequencer using a High Output Flowcell for 75bp single reads (Illumina).

*BCL to FASTQ conversion:* BCL files were converted to FASTQ files using bcl2fastq Conversion Software version v2.20.0.422 (Illumina).

*Raw data processing and quality control:* Raw data were processed using nfcore/rnaseq (version 1.5dev; National Genomics Infrastructure at SciLifeLab Stockholm, Sweden). Sequenced reads were trimmed for adaptor sequence (Trim Galore! 0.4.4 dev), the reads were aligned (STAR2.5.3a_modified), assigned and counted (featureCounts1.6.1). MultiQC report was generated with MultiQC v1.5. Genome reference and annotation data: GENCODE.org (*Mus musculus*; GRCm38.p6; release M17).

*Normalization and differential expression analysis:* Normalization and differential expression analysis was performed with DESeq2 (Galaxy Tool Version 2.11.40.2).

All sequencing data were deposited at the GEO data base, accession number GSE147666.

### 2.8 Bioinformatics and statistical analysis

The normalized RNA reads were used to generate volcano plots (Qlucore Omics Explorer software). GraphPad Prism was used for statistical analyses. One-way ANOVA followed by Tukey's multiple comparisons test was done when analyzing three or more data sets. Two-way ANOVA followed by Sidak's multiple comparisons test were used when analyzing two different groups of data sets.

## 3 Results

### 3.1 Differential transcriptome read-outs depend on cell isolation protocols

**Fig. 1:**
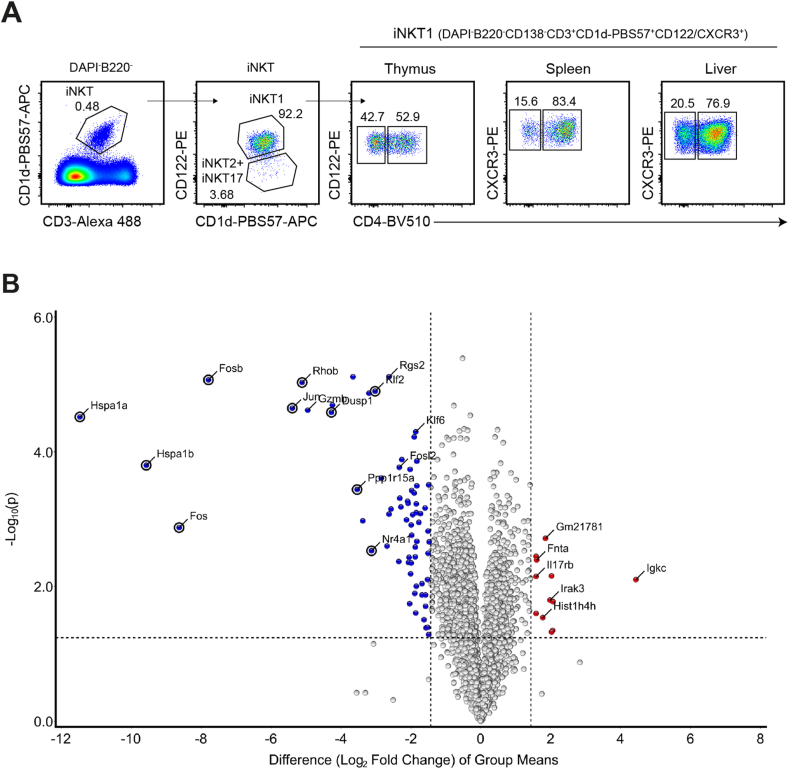
Genes expressed differentially by CD4^+^iNKT1 cells of liver and spleen. **(A)** Representative flow cytometry plots illustrating the gating strategy applied to identify iNKT subsets. Frequencies of iNKT cells in the corresponding gates are given. **(B)** Volcano plot illustrating genes upregulated (red dots) or downregulated (blue dots) in CD4^+^iNKT1 cells from spleen when compared to liver_H_ (fold change ≥3, p value ≤ 0.05). Data were collected from three independent RNA sequencing runs per cell type. Shown are 5887 genes with >300 RNA reads/gene in at least one of the three samples. (For interpretation of the references to color in this figure legend, the reader is referred to the Web version of this article.)

**Fig. 2:**
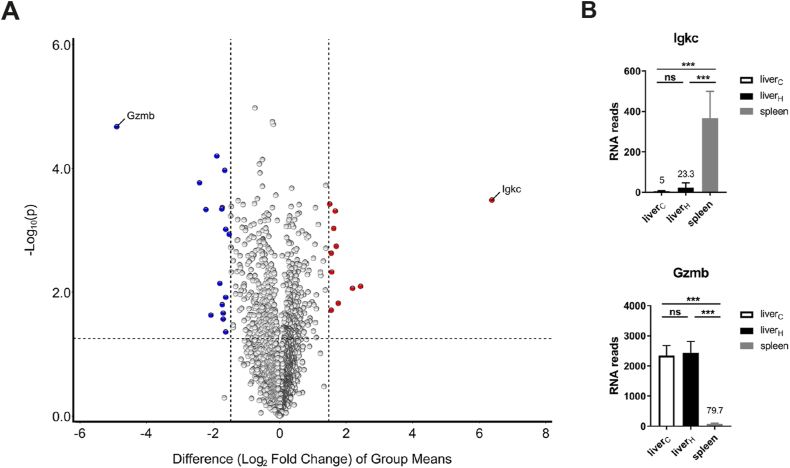
Mode of cell preparation influences RNA expression profile. **(A)** Volcano plot illustrating genes upregulated (red dots) or downregulated (blue dots) in CD4^+^iNKT1 cells from spleen when compared to liver_C_ (fold change ≥3, p value ≤ 0.05). Data were collected from three independent RNA sequencing runs per cell type. Shown are 6132 genes with >300 RNA reads/gene in at least one of the three samples. **(B)** RNA reads (mean ± sd) of the indicated genes in CD4^+^iNKT1 cells from liver_C_, liver_H_ and spleen. Data source as in A. One-way ANOVA followed by Tukey's multiple comparisons test were performed in B. ***p < 0.001, ns: not significant. (For interpretation of the references to color in this figure legend, the reader is referred to the Web version of this article.)

**Fig. 3:**
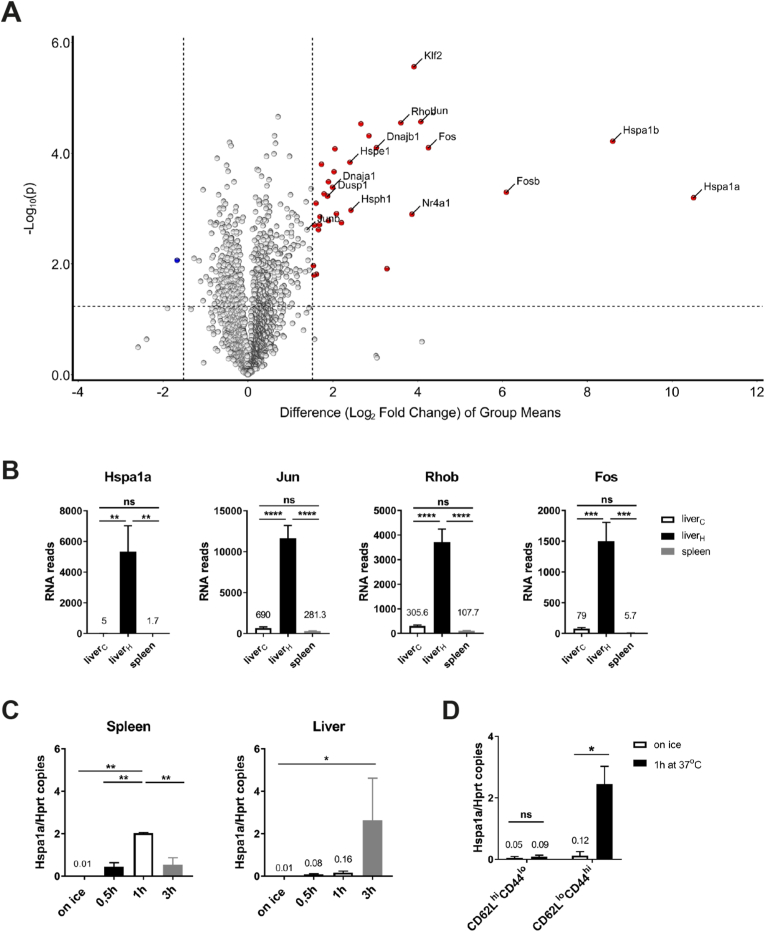
Temperature is a main factor influencing expression of stress genes. **(A)** Volcano plot illustrating genes up-regulated (red dots) or down-regulated (blue dots) in CD4^+^iNKT1 cells from liver_H_ when compared to liver_C_ (fold change ≥3, p value ≤ 0.05). Data were collected from three independent RNA sequencing runs per cell type. Shown are 6078 genes with >300 RNA reads/gene in at least one of the three samples. **(B)** RNA reads (mean ± sd) of the indicated genes in CD4^+^iNKT1 cells from liver_C_, liver_H_ and spleen. Data source as in Fig. 1, Fig. 2A. **(C)** Determination of *Hspa1a/Hprt* copies by RT-PCR in RNA samples of CD4^+^iNKT1 cells obtained in the cold from spleen or liver (samples on ice) and following incubation at 37 °C for 0.5, 1 and 3 h. **(D)** Determination of *Hspa1a/Hprt* copies by RT-PCR in RNA samples of CD62L^hi^CD44^lo^ and CD62L^lo^CD44^hi^ CD4^+^T cells (DAPI^−^CD8^-^B220^−^CD25^−^tet^-^) obtained from spleen and following incubation at 37 °C for 1 h. Data were collected from three independent experiments where the cells were pooled from 4 to 6 BL6 mice prior to sorting in (A) and (B), from 2 to 3 independent experiments where the cells were pooled from 2 to 4 BL6 mice in (C) and from 2 independent experiments analyzing 2 BL6 mice in (D). One-way ANOVA followed by Tukey's multiple comparisons test were performed in (B) and (C). Two-way ANOVA followed by Sidak's multiple comparisons test was performed in (D). *p < 0.05, **p < 0.01, ***p < 0.001, ****p < 0.0001, ns: not significant. (For interpretation of the references to color in this figure legend, the reader is referred to the Web version of this article.)

iNKT cells reside in virtually all tissues and organs of mice and human. Therefore, we were interested to investigate in more detail the tissue-specific phenotype of BL6 iNKT cells. To begin with, we performed a comparative RNA analysis of CD4^+^iNKT1 cells because they represent the most wide-spread subclass of iNKT cells in BL6 mice (Fig. 1A [16]). In these experiments, single cell suspensions of liver cells were prepared using a standard protocol utilizing Protein/DNA-degrading enzymes and lymphocyte enrichment by Percoll gradient centrifugation (as described in Materials and Methods). In contrast, spleen cells suspensions were generated as usual by simply mashing organ pieces through a nylon mesh in the cold. All subsequent steps (cell sorting, RNA isolation and sequencing) were performed identically for both sets of cells. When the transcriptomes of splenic and liver CD4^+^iNKT1 cells were compared, a moderate number of genes displayed a significant differential expression pattern (colored dots: > 3-fold difference, p ≤ 0.05) (Fig. 1B). Only *Igkc* coding the immunoglobulin kappa light chain was expressed distinctly stronger by spleen cells albeit at overall low levels (21.6-fold, 366.3 ± 76.9 RNA reads). Possibly, this was caused by a small cross-contamination of B cells even though care was taken during cell sorts by including B cell markers (B220, CD138). In contrast, the set of genes showing a liver-biased expression was larger. In addition, there was a series of genes expressed strongly and almost exclusively in liver cells. Strikingly, most of these genes are known to be involved in cellular stress response (bold dots in Fig. 1B): members of the family of heat shock genes (*Hspa1a*, *Hspa1b*) [17], of the family of Fos/Jun transcription factors (*Fos*, *Fosb*, *Jun*) [18] and others (*Rhob*, *Dusp1*, *Pppr1r15a*, *Nr4a1*, *Klf2*) [[19], [20], [21], [22], [23]]. Therefore, we questioned whether this finding reflects a genuine trait of the liver cells. To investigate this, we included RNA samples derived from liver CD4^+^iNKT1 cells that were prepared similar to spleen cells omitting enzymatic treatment. These liver cells were addressed as liver_C_ (C = cold) to emphasize the contrast to cells that were obtained following enzymatic digestion at 37 °C. The latter will be named liver_H_ (H = hot) cells from now on. Indeed, only the expression of two genes (*Igkc* and *Gmzb*) remained strongly biased when replacing liver_H_ by liver_C_ cells in the volcano plots (compare Figs. 1B and 2A). Fig. 2B depicts the actual read counts for these two genes. Interestingly, thymic iNKT1 cells express granzymes (*Gzma* and *Gzmb*) in an age dependent manner [24] suggesting that iNKT1 cells modulate their functionality in an age and organ specific manner. Apart from this, however, only very few genes displayed a moderate organ specific expression pattern (Fig. 2A). Finally, Fig. 3A shows the direct comparison of the transcriptomes of liver_H_ and liver_C_ CD4^+^iNKT1 cells. Virtually all genes expressed with a bias more than three-fold can be found among liver_H_ cells, i.e. their expression was induced or upregulated due to the mode of cell isolation. Fig. 3B and Supplementary Table S1 show the expression of some selected genes. When the study was extended to include liver cells from BALB/c animals, a pretty similar pattern emerged (Supplementary Fig. S1 and Supplementary Table S1) indicating that the observed stress response is not strain specific.

Moreover, we investigated how the two applied isolation protocols affected the yield and subset composition of the BL6 cells isolated from liver and spleen (Supplementary Fig. S2). In both organs, isolation in the cold resulted in higher yields of iNKT1 cells (see also section 3.3). In contrast, yields of tet^−^TCRβ^+^T cells of spleen but not liver were slightly influenced by the isolation procedure. With regard to the iNKT cell subset composition, we observed a moderate shift at the expense of iNKT1 cells in liver and spleen (Supplementary Fig. S2B).

### 3.2 Increased temperature promotes stress response

Most likely, the temperature during cell isolation represents a critical parameter. To test this, CD4^+^iNKT1 cell were isolated from spleen and liver under “cold” conditions as described above and incubated at 37 °C for up to 3 h. Expression of *Hspa1a* was induced as early as 30 min into the incubation time yet the kinetics of induction varied between organs (Fig. 3C). We also isolated regular CD4^+^T cells from spleen at low temperatures and separated them into pools of CD62L^hi^ naïve cells, and CD62L^lo^ antigen-experienced cells. Upon incubation at 37 °C, only the antigen-experienced cells exerted *Hspa1a*-induction (Fig. 3D). These results indicate that the observed stress response is not restricted to iNKT cells yet it is not executed by resting naïve cells.

### 3.3 Increased temperature propels P2X7/ART2.2 activity

**Fig. 4:**
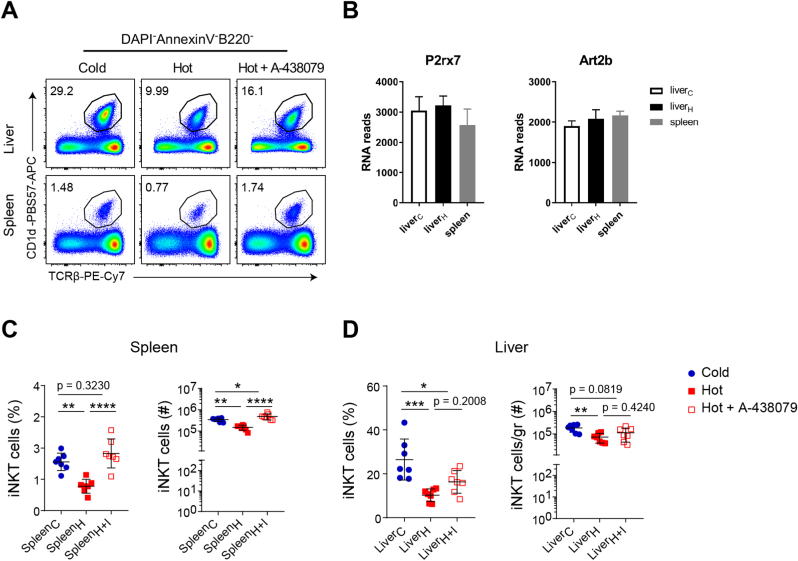
Activity of P2X7 affects yield and fitness of liver cells. **(A)** Representative flow cytometry plots illustrating iNKT cell frequencies among DAPI^−^Annexin V–B220^-^ cells in the liver and spleen of BL6 mice. **(B)** RNA reads (mean ± sd) of *P2rx7* and *Art2b* in CD4^+^iNKT1 cells from the indicated organs. Data were collected from 3 independent experiments where the cells were pooled from 4 to 6 BL6 mice. **(C**–**D)** iNKT cell frequencies and numbers in spleen and liver of BL6 mice. In order to obtain single cell suspensions, the organ was processed on ice (cold), at 37 °C (hot) or at 37 °C in the presence of a P2X7 inhibitor (hot + A-438079) as described in Materials and Methods. One-way ANOVA followed by Tukey's multiple comparisons test were performed in (B), (C) and (D). *p < 0.05, **p < 0.01, ***p < 0.001, ****p < 0.0001, ns: not significant.

During the experiments we noted that the recovery rate of iNKT cells depended on the mode of cell isolation (see also section 3.1). When sample preparation included steps at 37 °C, frequencies and yields of iNKT cells were lower when compared to samples avoiding increased temperatures (hot versus cold in Fig. 4A, C, D) probably because elevated temperatures promote cell apoptosis/necrosis. We observed that peripheral CD4^+^iNKT1 cells express high amounts of *P2rx7* and *Art2b* (Fig. 4B) confirming recent observations [25]. The receptor P2X7 (*P2rx7*) represents an ATP gated ion channel and has multiple functions in immune cells [26]. One of these would be induction of apoptosis in the presence of high concentrations of extracellular ATP. Importantly, ADP-ribosylation of the receptor by the ecto-enzyme ART2.2 (*Art2b*) augments tremendously the P2X7-mediated sensitivity to apoptosis. Thus, the expression of P2X7 and ART2.2 renders cells prone to apoptosis in the presence of already low concentrations of NAD^+^, the substrate required by ART2.2 to ADP-ribosylate P2X7. To elucidate the role of the P2X7/ART2.2-system in this context in more detail, cells from spleen were isolated and either kept in the cold or incubated for 1 h at 37 °C in cell culture medium in the presence or absence of the P2X7-inhibitor A-438079. The temperature-triggered decrease in iNKT cell frequencies as well as numbers were reverted completely by adding A-438079 to the medium (Fig. 4A, C). In contrast, the drug was much less effective when present during liver cell preparations (Fig. 4A, D). We observed a trend towards an increased frequency/yield of liver iNKT cells in the presence of A-438079 but the effects did not reach statistical significance. These findings are in line with an earlier report showing that applying a blocking anti-ART2.2 nanobody improves the yield and fitness of diverse T cell subsets [25]. However, the direct comparisons done here suggest that during cell preparations the P2X7/ART2.2-system contributed to cell losses in an organ-specific extent.

## 4 Discussion

The mode of cell preparation had a profound influence on the RNA sequencing results. Our results suggest that the transcriptomes represent a bona fide replica of genuine cellular activity only when the bioactivity of cells was “frozen” at the moment of their isolation. Although the expression of only a rather small set of genes was affected when tissue was processed at elevated temperatures, this had far-reaching implications. We observed a purposeful upregulation of heat shock genes. Enzymatic treatment of liver tissue induced strongly the expression of the two genes coding for HSPA1 [27]. A selective weaker but still significant upregulation of heat shock genes coding for proteins assisting HSPA1 function also occurred (*Hsph1*, *Dnaja1*, *Dnajb1*, *Hsp90aa1*, colored in Supplementary Table S1) while the expression levels of other heat shock genes remained unchanged. This indicated that the cells initiated mainly a classical heat stress response (and just a rather moderate ER stress response), i.e. a massive production of chaperons protecting from damage [17]. Moreover, AP-1 expression is strongly upregulated by stress. In addition, stress can cause regulation of AP-1 activity by post-translational mechanisms [18]. Similarly, *Rhob*, *Klf2*, *Dusp1*, *Ppp1r15a*, and *Nr4a1*, respectively, belong to the set of genes that are stress sensitive. For example, KLF2 promotes a quiescent phenotype opposing cell migration and can inhibit AP-1 transcription factors [19,28]. In contrast, GADD34 (*Ppp1r15a*) prepares cells for post-stress activities by assisting in dephosphorylation of translation initiation factor eIF2α thereby reversing a stress induced inhibition of protein synthesis [23]. These results demonstrate that the CD4^+^iNKT1 cells responded by a concerted action to stress that may consist of heat or rapidly changing temperatures, shear forces or deprivation of cell contacts as well as soluble factors being part of their regular *in situ* environment. Possibly, such response is inevitable because it accompanies any experimental manipulation of tissue and can be suppressed only as long as the cells are kept at temperatures slowing down or inhibiting their biological re-activities. At any rate, our results suggest that such considerations must be included in the interpretation of experimental observations. This may be illustrated by the finding that genes discussed here such as *Fos*, *Fosb*, *Jun*, *Junb*, *Rhob*, *Dusp1*, *Dnaja1*, *Dnajb1*, *Hsp90aa1*, *Klf2*, and *Nr4a1*, respectively, were identified as signature genes distinguishing resident from circulatory lymphocyte subsets [14,29,30]. In order to identify such diagnostic genes, the transcriptomes of circulatory and tissue-resident T cell subsets were compared. These cells were isolated from lymphoid organs representing a source for circulatory cells and from tissues like intestine or skin harboring tissue resident cells. Whereas the former cells were obtained by methods comparable to those defined here as “cold”, the latter cells were isolated following enzymatic treatment of tissue at 37 °C. Thus, the conspicuous enrichment of stress response genes among the collection of markers expressed by resident cells might be at least in part due to the different procedures applied to isolate the cells of interest.

## Declaration of competing interest

The authors declare no competing financial interests.
