# Supplementary material for: The impact of stress on the transcriptomic signature of iNKT1 cells
Source: Biochem Biophys Rep. 2021 Oct 29;28:101163. doi: 10.1016/j.bbrep.2021.101163 (PMC8570944; doi:10.1016/j.bbrep.2021.101163)
Supplement: Multimedia component 1 [file mmc1.docx]

Supplementary Table 1: List of heat shock genes

Listed are mainly members of the HSP70 family. Shown are only genes expressed at levels > 150 reads in liver (Lv) CD4^+^iNKT1 cells. Same source as in Fig. 1B, 2A and Supplementary Fig. 1A. Shaded areas denote genes with a stress sensitive expression as described in the text. Red: strong induction, lack of expression in cold; yellow: more moderate induction but already expressed in cold. Given are also the reads for *Dnaja1* and *Dnajb1* coding for members of the HSP40 protein family that act as co-chaperons of HSP70 proteins. *Hsp90aa1* codes for HSP90a, an inducible member of the HSP90 chaperon family. Values for spleen samples are shown for comparison.

| Gene | BL6  Spleen | BL6 Lv/cold | BL6 Lv/hot | BALB/c  Lv/cold | BALB/c Lv/hot | Information  (UniProt.org) |
| --- | --- | --- | --- | --- | --- | --- |
| *Hspa1a* | 3  0  2 | 0  4  11 | 6,764  3,472  5,766 | 27  20 | 4,509  4,987  7,933 | Chromosome 17  Chaperone  Stress protect |
| *Hspa1b* | 3  11  12 | 11  26  11 | 6,498  3,989  6,791 | 30  59 | 4,793  4,517  8,684 | Chromosome 17  Chaperone  Stress protect |
| *Hspa4* | 2,368  2,017  2,232 | 2,448  2,203  2,472 | 2,766  2,744  3,172 | 1,838  2,431 | 2,460  2,376  3,115 | Chromosome 11 Chaperone |
| *Hspa5* | 7,598  7,442  7,920 | 6,304  7,244  7,656 | 9,382  8,407  8,048 | 7,389  6,827 | 8,053  9,123  9,469 | Chromosome 2  ER* chaperone, BiP |
| *Hspa8* | 8,128  11,084  12,856 | 7,991  13,857  13,900 | 25,778  17,339  20,059 | 6,414  6,622 | 11,561  13,967  18,692 | Chromosome 9  Chaperone  Stress protect |
| *Hspa9* | 2,062  1,712  1,914 | 2,019  1,746  2,008 | 1,823  2,224  2,379 | 1,928  1,999 | 2,299  2,376  2,800 | Chromosome 18  Chaperone  mitochondrial |
| *Hsp13* | 308  376  381 | 215  318  314 | 388  238  318 | 433  339 | 465  618  288 | Chromosome 16  ER* Chaperone |
| *Hsp14* | 416  458  419 | 399  468  492 | 450  429  436 | 465  415 | 426  510  429 | Chromosome 2  Part of Ribosome-associated complex |
| *Hsph1* | 756  1,081  831 | 894  927  1,235 | 7,037  3,923  5,748 | 599  1,136 | 6,078  8,109  10,065 | Chromosome 5  NEF** for HSPA1 |
| *Hyou1* | 1,611  1,747  1,378 | 1,718  1,717  1,538 | 2,069  1,865  1,876 | 1,286  1,760 | 1,343  1,537  1,728 | Chromosome 9  GRP170; hypoxia induced upregulation |
| *Dnaja1* | 641  680  893 | 671  807  864 | 3,509  2,513  3,331 | 1,117  733 | 3,639  3,946  4,238 | Co-chaperon binding to HSPA1 |
| *Dnajb1* | 437  337  360 | 683  931  939 | 7,939  6,159  6,738 | 1,035  818 | 7,540  6,660  9,273 | Co-chaperon binding to HSPA1 |
| *Hsp90aa1* | 2,074  2,092  1,865 | 2,651  2,990  3,254 | 11,732  7,370  10,085 | 2,634  2,905 | 7,329  12,956  18,145 | Builds a chaperon complex with HSPA1 |

*: Endoplasmic Reticulum

**: Nucleotide-exchange factor promoting ADP release
