# Supplementary material for: The impact of stress on the transcriptomic signature of iNKT1 cells
Source: Biochem Biophys Rep. 2021 Oct 29;28:101163. doi: 10.1016/j.bbrep.2021.101163 (PMC8570944; doi:10.1016/j.bbrep.2021.101163)
Supplement: Multimedia component 2 [file mmc2.pdf]

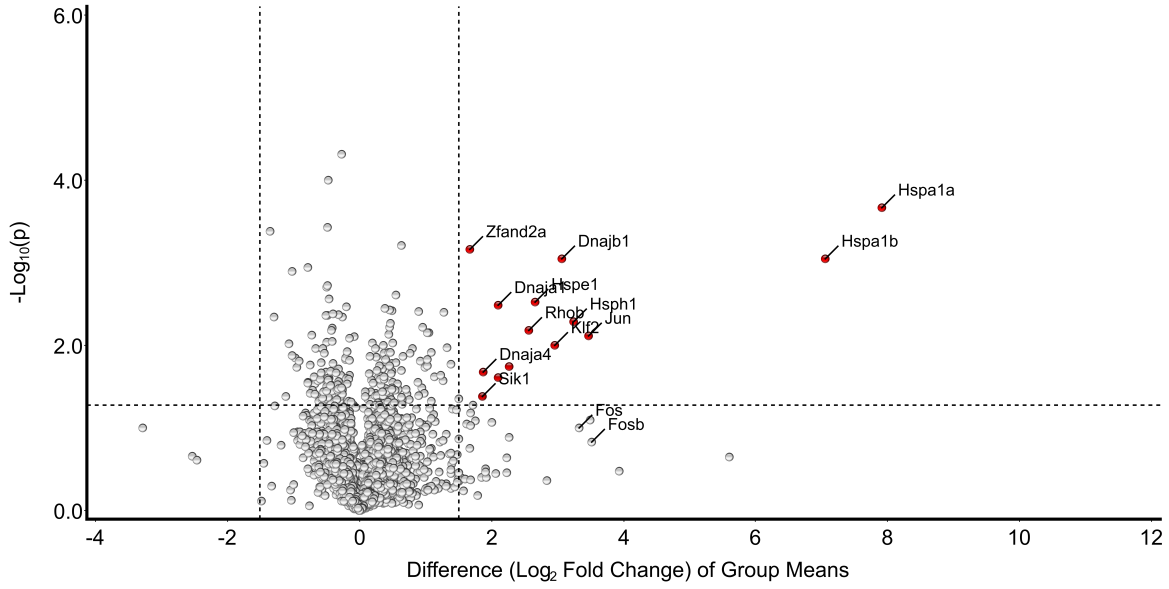

Supplementary Figure 1

Volcano plot illustrating genes upregulated (red dots) or downregulated (blue dots) in CD4^{+}iNKT1 cells from liver$_{H}$ when compared to liver$_{C}$ (fold change ≥3, p value ≤0.05). Data were collected from two (liver$_{C}$) or three (liver$_{H}$) independent RNA sequencing runs per cell type where the cells were pooled from 4-6 BALB/c mice. Shown are 5641 genes with >300 RNA reads/gene in at least one of the samples.
