# Supplementary material for: The impact of stress on the transcriptomic signature of iNKT1 cells
Source: Biochem Biophys Rep. 2021 Oct 29;28:101163. doi: 10.1016/j.bbrep.2021.101163 (PMC8570944; doi:10.1016/j.bbrep.2021.101163)
Supplement: Multimedia component 3 [file mmc3.pdf]

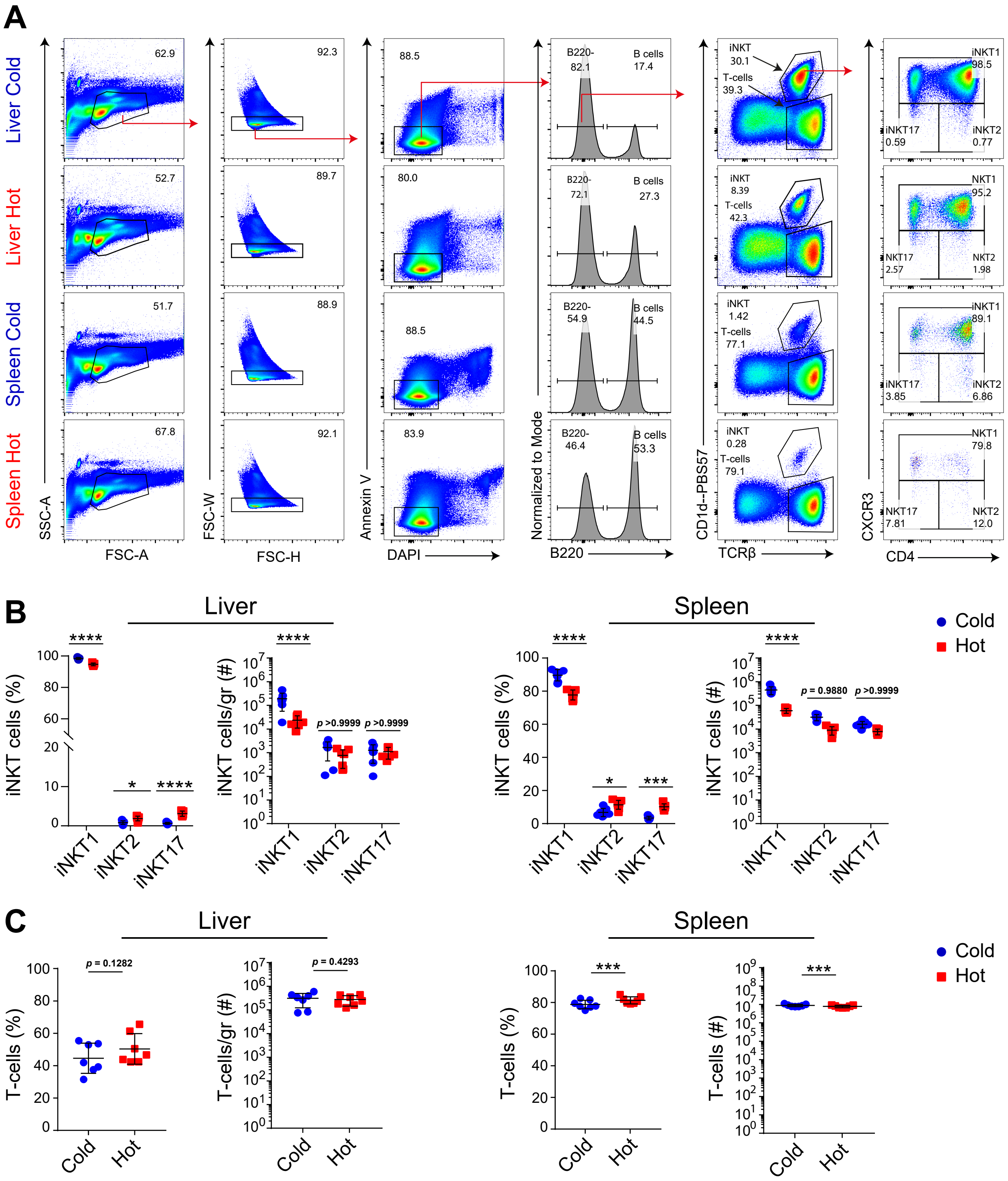

Supplementary Figure 2

Mode of cell preparation affects the yield of iNKTcells subsets. **(A)** Representative flow cytometry plots illustrating the frequencies of T cells, and the different subsets of iNKTcells (iNKT1, 2 and17) in the spleen and liver of BL6 mouse following different organ processing methods. Isolated organs were processed either on ice (cold) or at 37ºC (hot) as described in Materials and Methods. **(B-C)** Frequencies and numbers of T cells, iNKT1, 2 and, 17 cells in the spleen and liver of BL6 mice. Data is quantified according to the gating strategy displayed in **(A)**. One-way ANOVA followed by Tukey's multiple comparisons test were performed in **(B)** and **(C)**. *p<0.05, ***p<0.001, ****p<0.0001.
